# A large-scale perspective on stress-induced alterations in resting-state networks

**DOI:** 10.1038/srep21503

**Published:** 2016-02-22

**Authors:** Adi Maron-Katz, Sharon Vaisvaser, Tamar Lin, Talma Hendler, Ron Shamir

**Affiliations:** 1 Functional Brain Center, Tel Aviv Sourasky Medical Center, 6 Weizmann Street, Tel Aviv 64239, Israel; 2 Physiology and Pharmacology Department, Sackler Faculty of Medicine, Tel Aviv University, Tel Aviv 69978, Israel; 3 Blavatnik School of Computer Science, Tel-Aviv University, Tel Aviv 69978, Israel; 4 Sagol School of Neuroscience, Tel Aviv University, Tel Aviv 69978, Israel; 5 School of Psychological Sciences, Tel Aviv University, Tel Aviv 69978, Israel

## Abstract

Stress is known to induce large-scale neural modulations. However, its neural effect once the stressor is removed and how it relates to subjective experience are not fully understood. Here we used a statistically sound data-driven approach to investigate alterations in large-scale resting-state functional connectivity (rsFC) induced by acute social stress. We compared rsfMRI profiles of 57 healthy male subjects before and after stress induction. Using a parcellation-based univariate statistical analysis, we identified a large-scale rsFC change, involving 490 parcel-pairs. Aiming to characterize this change, we employed statistical enrichment analysis, identifying anatomic structures that were significantly interconnected by these pairs. This analysis revealed strengthening of thalamo-cortical connectivity and weakening of cross-hemispheral parieto-temporal connectivity. These alterations were further found to be associated with change in subjective stress reports. Integrating report-based information on stress sustainment 20 minutes post induction, revealed a single significant rsFC change between the right amygdala and the precuneus, which inversely correlated with the level of subjective recovery. Our study demonstrates the value of enrichment analysis for exploring large-scale network reorganization patterns, and provides new insight on stress-induced neural modulations and their relation to subjective experience.

Acute stress has been shown to have dramatic effects on the way our brain functions, which can be viewed as a strategic resource reallocation to functions that are required when facing a threat such as promoting arousal^1^,^2^ and memory encoding of stressful experiences^3^,^4^,^5^, at the cost of higher cognitive functions^6^,^7^,^8^. However, while the neural basis of the stress response at the time of induction has been widely investigated, less is known about the neural processes that underlie successive recovery in humans. Characterizing individual variability in recovery from stress is of a particular interest since it has been associated with several stress-related psychopathologies, including Post Traumatic Stress Disorder (PTSD) and depression^9^,^10^.

One approach to study post-processing of prior events, such as stress, is by inspecting the spontaneous neural activity that takes place during rest after the event occurred. It has been suggested that this post-processing supports prior experience consolidation^11^,^12^,^13^, and thus, may play a central role in regaining mental and physiological homeostasis, and is expected to involve large scale brain network reorganization^14^,^15^,^16^. Accordingly, using post-stress resting-state functional magnetic resonance imaging (rsfMRI) to investigate network reorganization following stress may provide a vital insight into the large-scale neural mechanism that underlies affective recovery from acute stress.

Few previous fMRI studies investigated changes in resting-state functional connectivity (rsFC) following acute stress^17^,^18^,^19^. For example, van Marle *et al.* reported increased amygdala rsFC immediately following acute stress with anterior cingulate cortex, anterior insula, and a dorso-rostral pontine region^18^. In another study Veer *et al.* reported increased amygdala rsFC with the posterior cingulate cortex (PCC), precuneus and medial prefrontal cortex an hour following stress, suggesting that these effects could be related to top-down control of the amygdala and consolidation of self-relevant information following a stressful event^19^. Lastly, Vaisvaser *et al.*^17^ examined changes in rsFC patterns seeded at the PCC and hippocampus. Unlike the two aforementioned studies, here rsFC alterations were examined with respect to the pre-stress resting period. Immediately after stress induction several rsFC changes were reported including altered coupling within the default mode network (DMN) and between hippocampus and amygdala. Notably, these studies used a hypothesis-driven fMRI analysis approach, exploring connectivity changes involving one or few predefined seed regions. Alongside the clear statistical advantages of such a seed-based approach, lies the disadvantage of revealing only that fraction of the actual phenomena that involves the preselected seed, and possibly missing other relevant findings, which can be identified using a data-driven approach. This disadvantage is even more crucial when investigating stress, which is known to induce large-scale network re-organization, as demonstrated by Hermans *et al.* who used group independent component analysis (ICA) in combination with inter-subject correlation analysis to identify large-scale stress-related FC changes induced during exposure to fear-related movie clips. They reported an increase in interconnectivity within a salience network, which positively correlated with the magnitude of subjective stress response^8^. This network included cortical (frontoinsular, dorsal anterior cingulate, inferotemporal, and temporoparietal) and subcortical (amygdala, thalamus, hypothalamus, and midbrain) regions. Accordingly, it has been suggested that exposure to acute stress prompts the recruitment of a salience network, at the expense of a fronto-parietal executive control network involving dorso- frontal and parietal areas, and that this resource allocation is reversed after stress subsides^15^. Nonetheless, large scale alterations after exposure to stress require further identification and deeper characterization.

In this study we aimed to gain a broader perspective on rsFC modulations following acute social stress, and examine their correspondence to individual subjective experience. To this end we adopted a data-driven approach for analyzing rsfMRI data recorded from healthy male subjects before and after performing the arithmetic task from the well-established Trier Social Stress Test^20^ (TSST), adapted to the scanner^17^,^21^. In addition, in order to study the relation between stress-induced rsFC modulations and subjective experience of recovery, we divided our participants according to their reported stress sustainment.

Data analysis was conducted using a fine-grained predefined functional parcellation^22^ that allowed dimensionality reduction on one hand, while maintaining a relatively coherent per-parcel blood oxygenation level-dependent (BOLD) signal on the other hand. In this parcellation most anatomic structures are covered by more than one parcel. This redundancy in regional representation along with the expected large-scale effect of stress induction may lead to a large number of identified changes even after controlling for type-I error. In such cases an additional means is required in order to pinpoint the most dominant rsFC changes. To this end we applied a second-level analysis termed *enrichment analysis*, which is commonly used in the field of Bioinformatics for interpreting a large number of noisy results in a statistically sound manner^23^,^24^. In the current study enrichment analysis was conducted based on parcel anatomic positions, seeking pairs of lobes that were over-represented (i.e. significantly more prevalent than would be expected by chance) in the set of identified modulations.

We hypothesized that using a whole-brain data-driven approach would reveal a large-scale effect of stress-induced rsFC modulations, which corresponds to changes in the subjective experience of stress. We expected some of these changes to involve rsFC that had been previously associated with stress reactivity, such as connections within the salience network and executive control network, as suggested in^15^, and rsFC previously associated with post-stress processing, e.g. within the DMN or between the DMN and limbic regions^17^,^19^. Furthermore, we expected some of these stress-induced changes to be sensitive to inter-individual differences in subjective stress recovery measured 20 minutes after the stress eliciting experience.

## Results

### Behavioral and physiological indications of stress

**Figure 1:**
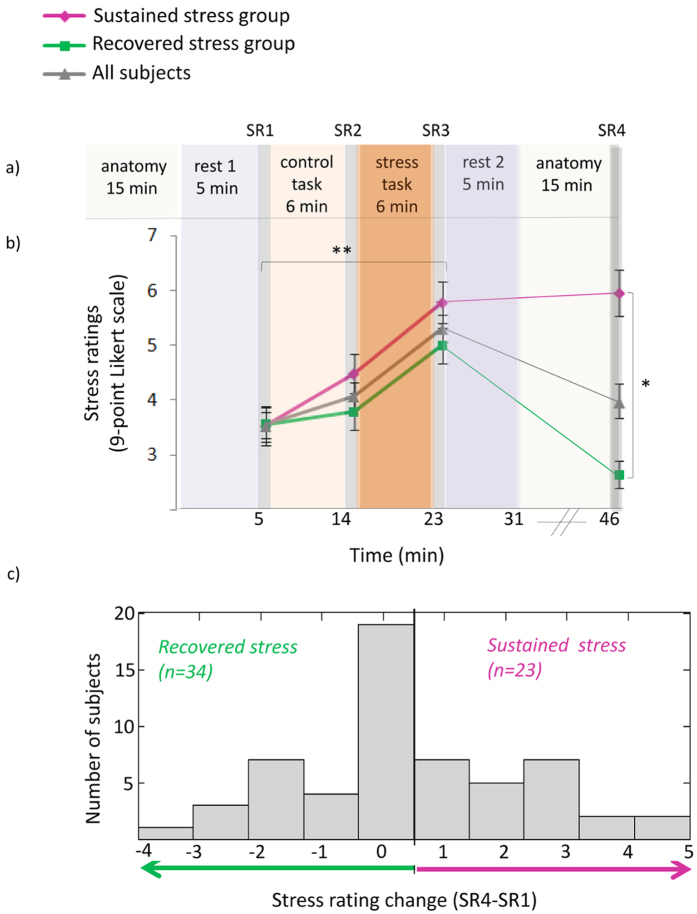
Psychological response to stress on experimental timeline. Subjective ratings of stress (**b**) are presented in reference to the time course of the experiment (**a**). Time 0 indicates the start of the first rest condition. The orange columns represent control and stress tasks (6 min each), violet columns represent ‘rest’ conditions (fixation, open eyes, 5 min) and light gray columns represent anatomical scans (15 min each). Between scans (dark gray columns), subjective ratings of stress and salivary cortisol samples [SR(1–4)] were collected. Bars indicate standard error. (**c**) Group partition marked on the distribution of change in subjective stress ratings. *p < 0.005, **p < 0.0001 – Tukey’s HSD post hoc.

Stress elicitation was indicated across participants by behavioral as well as physiological measures. Specifically, a main effect of time was found for subjective ratings of stress [F(3,165) = 25.76, p < 0.0001] Tukey’s honest significant difference (HSD) post-hoc analyses revealed an increase in ratings in response to stress (SR3) as compared to the two previous measures (SR1 and SR2, both p-values < 0.0001), and a decline to baseline following “rest2” (SR4, p < 0.0001, Fig. 1b). A main effect of time was found also in HR (beats per minute) [*F*_(3,123)_ = 36.35, p < 0.0001; 42 participants with a reliable R peak signal were included in this analysis]; Tukey’s HSD post-hoc analyses revealed an increase in HR in response to stress, as compared to pre-stress conditions (p < 0.005), and a decrease to initial levels during the second rest period (p < 0.0001). For salivary cortisol, a significant main effect of time was identified, with a peak in cortisol level in the final sample (SR4) as compared to post “rest1” sample [F(3,159) = 2.66, p < 0.05; 54 participants with sufficient saliva samples were included in this analysis]; Tukey’s HSD post-hoc analyses revealed a marginally significant peak in cortisol level in the final sample (SR4) as compared to post “rest 1” sample (SR1, p = 0.08).

Stress sustainment versus recovery group division: Out of 57 participants, 23 demonstrated elevated reported stress levels 20 minutes post stress induction (i.e. SR4-SR1 > 0), and were thus assigned in the current study to the “sustained stress” group. The rest of the subjects (n = 34) were assigned to the “recovered stress” group. For the “recovered stress” group a significant decline in stress ratings was identified 20 minutes following stress-induction (SR4) relative to ratings immediately after stress induction (SR3, Tukey’s HSD p < 0.0001; Fig. 1b). This decline was not evident in the “sustained stress” group. The SR4-SR1 value distribution is shown in Fig. 1c.

Notably, the analysis of cortisol at the 4 time points as well as HR (bits per minute) by subjective stress groups (Stress sustainment versus recovery) revealed no significant interaction between group and time [F(3,156) = 0.16, p > 0.9 and F(3,120) = 0.204, p > 0.1 respectively].

### Stress-induced rsFC alterations

**Figure 2:**
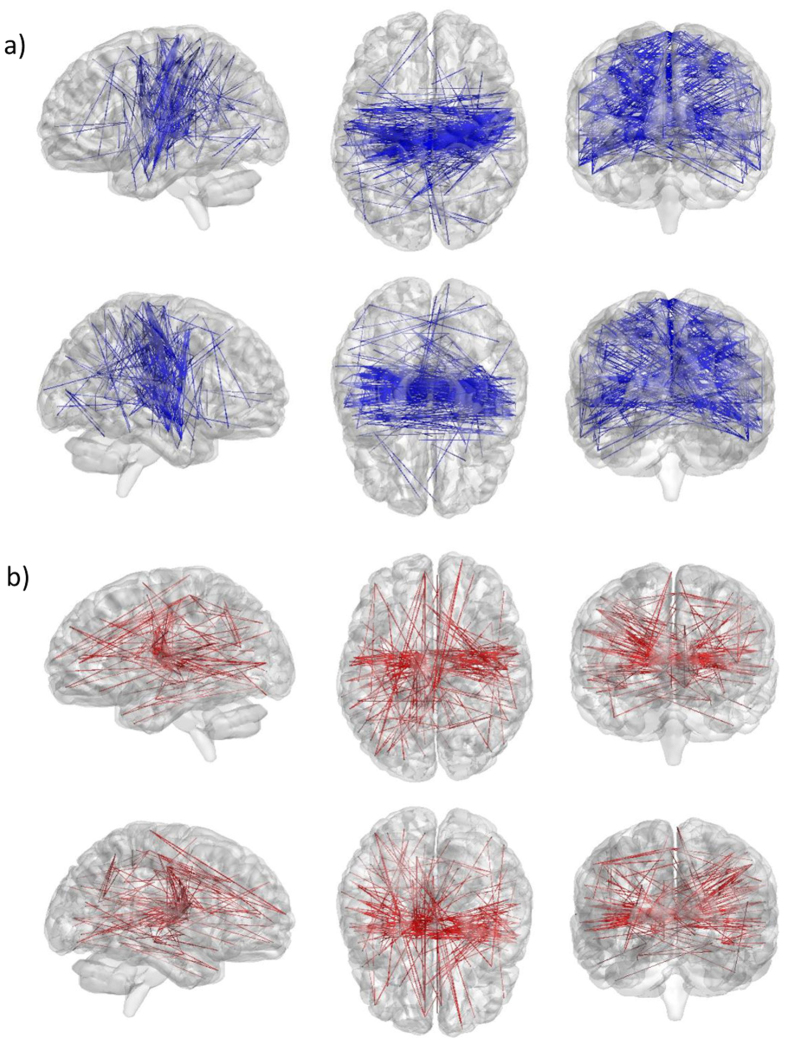
Significant rsFC changes following stress. Using FDR of 0.05, 490 parcel-pairs demonstrated a significant ∆FC between “rest1” and “rest2”. Of these, 301 demonstrated rsFC decrease (**a** - shown in blue) and 189 demonstrated rsFC increase (**b** - shown in red). Top and bottom rows of (**a**) and (**b**) both show sagittal, axial and coronal views, but differ in viewpoint (e.g. sagittal-left vs. sagittal-right). Visualization was generated using Brain Net Viewer^53^.

In order to identify post-stress rsFC alterations across the brain, we conducted a univariate statistical analysis on the Fisher-transformed cross-correlation matrices. This was done by subtracting the “rest1” matrix from the “rest2” matrix, and then applying a one-sample t-test on the resulting FC change value (denoted ∆FC) of each parcel pair. A significant rsFC change (FDR q < 0.05) was identified in 490 out of 106,953 possible parcel-pairs. Of these, 189 pairs demonstrated rsFC increase and 301 demonstrated rsFC decrease. Pairs are presented as connections/edges on a 3D brain image in Fig. 2. This large-scale effect required a second-level analysis in order to highlight the main findings. To this end, we conducted enrichment analysis.

**Figure 3:**
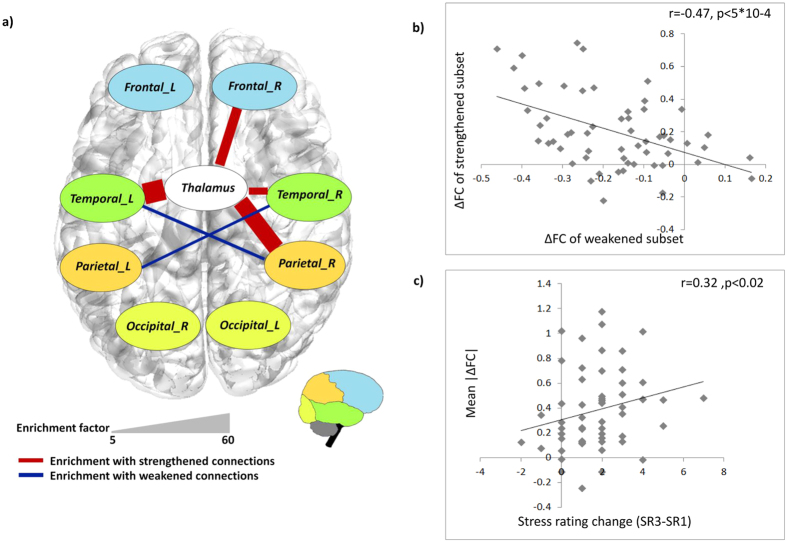
Enrichment analysis results. (**a**) Lobe distribution of connections that demonstrated significant ∆FC from “rest1” to “rest2”. Each node corresponds to a lobe in the analysis. An edge indicates significant over-representation of the corresponding lobe pairs in the set of strengthened connections (red) and the set of weakened connections (blue). Edge width reflects the enrichment factor (EF) of the identified connections. (**b**) A scatter plot presenting the ∆FC across strengthened subset against ∆FC across weakened subset of parcel-pairs that were involved in the identified enrichments. Each spot shows the two values for one subject. A significant anti correlation is identified (Pearson r = −0.47, p < 0.0005, n = 57). (**c**) A scatter plot presenting the mean ∆FC across all parcel-pairs that were involved in the identified enrichments against the (SR3-SR1) change in subjective stress rating. Each spot shows the two values for one subject. A significant correlation is identified (Spearman r = 0.32, p < 0.02, n = 57).

Using the lobe and laterality annotation of each parcel, we searched for pairs of annotations that were significantly over-represented (i.e. “enriched”) in the set of connections identified as affected by the stress task across all subjects. Enrichment analysis was applied separately on the set parcel-pairs demonstrating rsFC increase (i.e. “strengthened set”) and on the set parcel-pairs demonstrating rsFC decrease (i.e. “weakened set”). Results are summarized in Table 1 and illustrated in Fig. 3a. The strengthened set was found to be enriched with thalamo-frontal (right), thalamo-temporal (bilateral) and thalamo-parietal (right) connections, while the weakened set was found to be enriched with cross-hemispheral temporo-parietal connections, including regions of the inferior, middle and superior temporal gyri along with regions of the pre- and post central gyri and the superior and inferior parietal lobule. Supplementary Table S1 contains information on enrichment-inducing pairs (i.e. parcel-pairs that were both modulated by the task and link lobe pairs that were found to be enriched).

### Relation between stress-induced rsFC changes and stress indications

Examination of the relation between the mean ∆FC magnitude across all 103 enrichment-inducing pairs and the reported change in stress immediately after induction (i.e. SR1 vs SR3) across all subjects, revealed a significant positive correlation (Spearman r = 0.32, p < 0.02, n = 57; Fig. 3c). When conducting the same test separately for the 46 parcel-pairs involved in strengthened rsFC enrichment (the “strengthened subset”, Table 1) and the 57 parcel-pairs involved in weakened rsFC enrichment (the “weakened subset”), a significant correlation was found for the strengthened subset (Spearman r = 0.265, p < 0.05, n = 57), but not for the weakened subset (Spearman r = −0.21, p = 0.115). Nevertheless, the mean ΔrsFC within the weakened subset was found to be significantly anti-correlated with the mean ΔrsFC within the strengthened subset (Pearson r = −0.47, p < 0.0005, n = 57; Fig. 3b). Notably, no association was found between the mean ∆FC magnitude across enrichment inducing pairs and the AUCi measure of cortisol increase (p > 0.5), nor was it associated with the “rest 1” to “rest 2” HR increase (p > 0.3).

**Figure 4:**
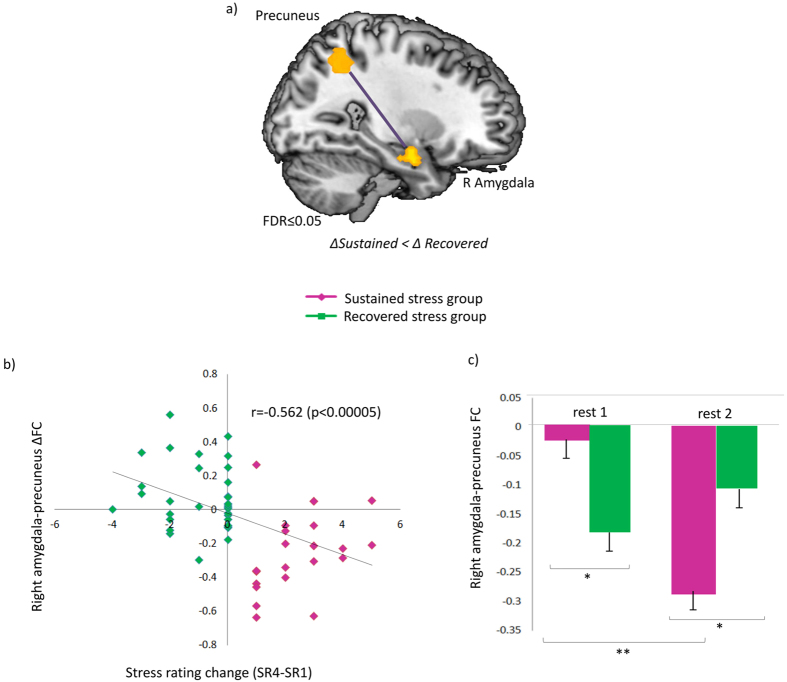
Results of inter-group rsFC change comparison. (**a**) A connection between the right amygdala and the precuneus that was identified in the inter-group ∆FC analysis. (**b**) A scatter plot presenting the right amygdala - precuneus ∆FC against the (SR4-SR1) change in subjective stress rating. Each spot shows the two values for one subject and is colored by group assignment (green – “recovered stress”, purple – “sustained stress”). A significant anti-correlation is identified (Spearman r = −0.562, p < 0.00005, n = 57) (**c**) right amygdala-precuneus rsFC patterns of “sustained stress” group (purple) and “recovered stress” group (green). Bars indicate standard error. *p < 0.001, **p < 0.0005.

In order to identify functional connections for which the change induced by stress was associated with affective stress sustainment, we used the SR4-SR1 stress-rating-based group partition (described above), and applied a two-sample t-test on ∆FC values of enrichment-inducing parcel-pairs. No significant inter-group difference was identified in ΔFC of any of the pairs separately (FDR q > 0.9). Additionally, there was no significant inter-group difference in the mean ΔFC magnitude of all 103 enrichment-inducing pairs (p > 0.6). Following this lack of association, we conducted a similar two-sample t-test on the entire set of 106,953 parcel-pairs in the data. Once again, an FDR procedure was used to correct for multiple hypothesis testing. Only one parcel pair demonstrated a significant inter-group difference in ∆FC between groups (with an FDR of 0.05). Parcels of the identified pair were anatomically mapped to the right amygdala and to the precuneus, based on parcel spatial centers (x = 6, y = −54, z = 48 and x = 27, y = −3, z = −21 respectively) (Fig. 4a).

We further examined the relationship between this amygdala-precuneus rsFC modulation and the longer term change in subjective stress ratings (SR4-SR1) across all subjects, and found a negative association between them (Spearman r = −0.526, p < 0.00005, n = 57, Fig. 4b). Additionally, a repeated measures ANOVA conducted on the corresponding Fisher-transformed rsFC values at both conditions (“rest1” and “rest2”), revealed an interaction between condition and group [F(1,55) = 32.6, p < 0.001]. Finally, Tukey’s HSD post-hoc analyses revealed that amygdala-precuneus rsFC was higher (less anti-correlated) among the “sustained stress” group than among the “recovered stress” group during “rest 1” (p < 0.05), and significantly declined following stress only among the “sustained stress” group (p < 0.001), leading to a significant inter-group difference also during “rest2” (p < 0.02). The means and standard deviation (in parenthesis) of “rest1” and “rest2” were −0.12 (0.24) and −0.18 (0.23) respectively, for all subjects, −0.024 (0.23) and −0.289 (0.24) for the “sustained stress” group and −0.18 (0.22) and −0.11 (0.2) for the “recovered stress” group. Results are shown in Fig. 4c.

The above group-based analysis was repeated for cortisol response-based groups in order to identify functional connections for which the change induced by stress was associated with cortisol increase. This analysis revealed a single parcel-pair which demonstrated a marginally significant inter-group difference in ∆FC (FDR q = 0.065). For further details see supplementary section.

## Discussion

In this work we conducted a data-driven investigation of stress-induced rsFC alterations and their correspondence to the subjective experience of stress, among a cohort of 57 healthy male participants. The induction of stress following the task was indicated by physiologic measures (namely HR and cortisol increase) as well as by subjective stress reports. However, intriguingly, no association was found between physiologic and behavioral measures. This result is in agreement with a recent review^25^ presenting studies that employ the TSST and related paradigms and point at a lack of correlation between the endocrine response and the level perceived stress, anxiety or negative affect^25^,^26^,^27^,^28^,^29^,^30^,^31^.

In line with our hypothesis, our analysis revealed a large-scale effect of rsFC modulations following acute social stress induction, which would not have been identified using a seed-based approach. Pinpointing the most significantly prevalent rsFC modulations, our enrichment analysis unraveled a pattern of decreased cross-hemispheral temporo-parietal connectivity along with increased thalamo-cortical (frontal, temporal and parietal lobes) connectivity. Importantly, as we predicted, these patterns of change in connectivity strength were associated with the change in subjective stress reports across subjects. Specifically a larger mean increase in reported stress immediately after the task was associated with a larger absolute rsFC change across all parcel-pairs forming both the strengthened and the weakened enriched connectivity alterations.

Our work extends previous studies investigating post-stress rsFC modulations in a hypothesis-driven manner^17^,^18^,^19^, by providing a broader unbiased picture. Identifying the thalamus as a central node of stress-induced rsFC increase is consistent with its known role in arousal regulation^32^ and in mediating the interaction of attention and arousal in humans^33^. Notably, the thalamus was found to be involved in post-stress rsFC alteration in our previous seed-based study, increasing its connectivity with the PCC (Vaisvaser *et al.*, 2013). Increased rsFC of the thalamus with several cortical regions including the Insula and IPL was also reported following fearful in comparison to neutral movies^16^.

In addition, a larger increase in thalamo-cortical rsFC was associated with a larger decrease in temporo-parietal rsFC, suggesting that both patterns are part of a joint mechanism of dominance-shift induced by acute stress. The identified pattern of stress-induced rsFC weakening involved regions of the inferior, middle and superior temporal gyri along with regions of the pre- and post central gyri and the superior and inferior parietal lobule. Most of these regions were reported to exhibit reduced BOLD activation in a within-subject analysis comparing high-stress task to a control task using a similar experimental paradigm^21^ and have been repeatedly reported to increase activity in attention-driven goal-directed tasks^34^,^35^,^36^,^37^,^38^.

These findings are in overall agreement with the recently suggested model by which exposure to acute stress prompts a reallocation of resources to a salience network, involving several subcortical regions including the thalamus, and several cortical regions in the frontal, temporal and parietal lobes, at the cost of an executive control network, involving dorsal frontal areas and dorsal posterior parietal areas^15^.

In addition to the above large-scale pattern of rsFC alterations, which were evident across subjects, we were interested in neural modulations that underlie inter-individual differences in the sustainment versus recovery of the subjective stress experience. Being sensitive to inter-individual differences, such modulations may not be detectable when looking across participants, i.e. in the first analysis. Thus, we conducted a second group-based analysis using the experience-based grouping. We identified a single modulation of rsFC between the right amygdala and the precuneus that differed between individuals with self-reported “sustained stress” and individuals with “recovered stress”. Importantly, this single statistically significant rsFC modulation was identified out of over 100,000 possible parcel-pairs without making any a-priori assumptions. The amygdala had been acknowledged as an important locus for integrating the various hormonal and neurotransmitter systems that are involved in consolidation following exposure to acute stress^4^. Moreover, previous evidence points to casual involvement of the right amygdala in generation of the subjective experience of fear and mark it as a potential therapeutic target in anxiety disorder^39^. The precuneus is a node of the DMN known to play a central role in a wide range of complex tasks, including self-referential processing and an experience of agency^40^. Abnormal precuneus activity and connectivity patterns have been previously reported in PTSD patients^41^,^42^,^43^,^44^,^45^. Importantly, spontaneous BOLD activity in the amygdala has been shown to be negatively correlated with the activity in the PCC and precuneus in healthy subjects^46^,^47^, and abnormal patterns of precuneus- amygdala rsFC have been previously reported in anxiety disorders^44^,^48^,^49^. In the current study we found a stress-induced enhancement of the amygdala -precuneus anti-correlation in “rest2” as compared to “rest1” only in individuals who reported a sustained stress experience, i.e., the “sustained stress” group. Furthermore, when accounting for inter-individual differences, we found that the extent of this single modulation predicted the level of affective recovery reported 20 minutes later across all subjects. Notably, an inter-group difference in rsFC of this connection was already evident during “rest 1”, with a lower level of anti-correlation among the “sustained stress” group, which predicted the pattern of subjective recovery following stress. These findings suggest that amygdala-precuneus rsFC may underlie the individual tendency and dynamics of subjective stress recovery.

In conclusion, using a statistically sound data-driven approach we were able to characterize stress-induced large-scale rsFC modulations, that were further associated with subjective experience. In addition, our group-based analysis pinpointed stress-induced rsFC change between right amygdala and precuneus as a neural predictor of affective recovery. This specific connection may serve as a potential biomarker and target for future treatment in stress-related disorders. We encourage future investigations of the stress-induced rsFC dynamics presented in the current research in other healthy populations and in psychopathology.

## Materials and Methods

### Participants

We used fMRI data reported in^17^, which was recorded from 61 healthy male subjects (age 19–22). Participants had no reported history of psychiatric or neurological disorders, no current use of psychoactive drugs, no family history of major psychiatric disorders, and no previous exposure to abuse during childhood and/or potentially traumatic events before entering the study. In addition, all participants had normal or corrected-to-normal vision and provided written informed consent. Experimental procedure and consent forms were approved by Tel Aviv Sourasky Medical Center Ethics Committee and conformed to the Code of Ethics of the World Medical Association (Helsinki Declaration). Of the 61, two individuals were excluded from the current analysis due to signal artifacts and two due to excessive head movements; therefore the final study group consisted of 57 participants.

### Experimental procedure

Each participant underwent a 65 minutes MRI scan that consisted of 6 phases: acclimation and anatomical scan (15 minutes), a rest condition (“rest1”, 5 minutes), control task (6 min), a social stress task (6 minutes), a second rest condition (“rest2”, 5 minutes) and another anatomical scan (15 minutes). Acute stress was induced using a serial subtraction arithmetic task^20^,^21^, fully described in^17^. Briefly, participants were instructed to continuously subtract 13 from 1022 for a period of 6 minutes, responding verbally, while monitored on-line by an experimenter. The stress task was preceded by a non-stressful condition of backward counting for a period of 6 minutes, without external monitoring. The experimental timeline is shown in Fig. 1a. During the rest conditions participants were instructed to keep their eyes open and stare at a fixation point. Psychological effect of stress (on a 9 point Likert scale) and salivary cortisol were evaluated at four time points: after the first rest scan (Stress Report 1; SR1), after the control task (SR2), right after the stress task (SR3) and 20 minutes after the stress task, following the second anatomical scan (SR4) (Fig. 1a). In addition, Electrocardiography (ECG) was recorded continuously during scanning via a BrainAmp ExG MRI-compatible system at a sampling rate of 5000 Hz, and used to extract heart-rate measure.

### fMRI data acquisition

Brain scanning was performed on a 3 T (GE, HDXt) MRI scanner with an 8-channel head coil. Functional imaging was acquired with gradient echo-planar imaging (EPI) sequence of T2^*^-weighted images (TR/TE/flip angle: 3000/35/90; FOV: 20 × 20 cm; matrix size: 96 × 96) in 39 axial slices (thickness: 3 mm; gap: 0 mm) covering the whole cerebrum.

### fMRI preprocessing and parcellation

fMRI data preprocessing was performed with SPM5 (Wellcome Department of Imaging Neuroscience, London, UK). It included correction for head movements via realignment of all images to the mean image of the scan using rigid body transformation with six degrees of freedom (subjects with movement above 2 mm were discarded), normalization of the images to Montreal Neurological Institute (MNI) space by co-registration to the EPI MNI template via affine transformation, and spatial smoothing of the data with a 6 mm FWHM. The first 6 images of each functional resting scan were excluded to allow for T2^*^ equilibration effects. Before further analysis, blood oxygenation level-dependent (BOLD) signals were filtered to low frequency fluctuations (0.01–0.08 Hz) using DPARSF toolbox^50^.

We used a whole brain functional parcellation reported in^22^, which was generated by applying a correlation-based clustering procedure on rsfMRI data recorded from 41 healthy subjects, and partitions the brain volume into 517 parcels. Parcels were masked to include gray matter voxels only using the WFU Pick Atlas Tool^51^,^52^, and 54 parcels that had less than 5 voxels in common with the gray matter mask were excluded, leaving 463 parcels. For each subject, average BOLD value across all gray matter voxels was calculated within each parcel at each time point of the two rest periods. These time series were used as the parcel’s signal. In order to reduce the effect of physiological artifacts and nuisance variables, the whole-brain mean signal, six motion parameters, cerebrospinal fluid, and white matter signals were regressed out of these parcel signals.

### Parcel-based functional connectivity analysis

**Figure 5:**
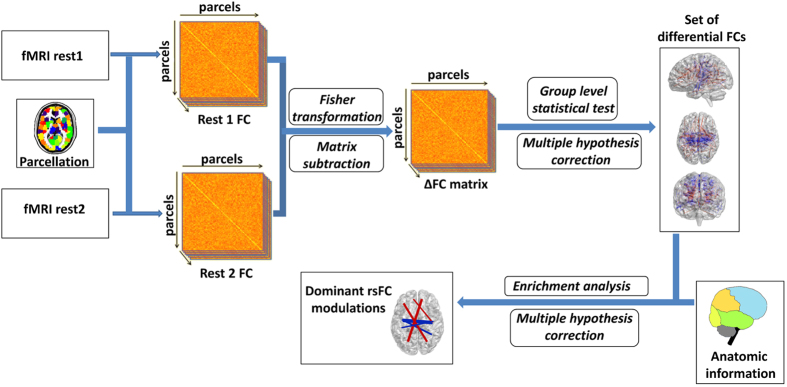
An illustration of the rsFC analysis steps. Following parcellation, cross-correlation matrices were calculated for each subject and resting-state session. A paired t-test was applied on the Fisher-transformed rsFC values to identify parcel-pairs for which rsFC changed significantly. An FDR procedure was used to correct for multiple testing. Next, anatomy-based enrichment analysis was used to characterize the identified changes in rsFC.

The procedures of rsFC analysis and statistical characterization are illustrated in Fig. 5. We used a univariate analysis approach, in which a model is fitted independently to each connection to assess evidence for experimental effects.

Level of rsFC between every two parcels was estimated by calculating the Pearson correlation coefficient between the corresponding signals. This was done for each subject and each rest condition separately. Correlation values were next Fisher transformed to better fit a normal distribution. FC level estimates of “rest1” were then subtracted from the corresponding estimates in “rest2”, resulting in a single FC change value (denoted ∆FC) for each pair of parcels and for each subject. To identify parcel-pairs that demonstrated significant rsFC change following the stress task, we applied a one-sample t-test on the ∆FC values of each pair across all subjects. To identify parcel-pairs that demonstrated a significant inter-group difference in ∆FC, we applied a two-sample t-test on the ∆FC values of each pair between groups.

### Statistical characterization of identified connections using enrichment analysis

rsFC modulations identified in the above univariate analysis were statistically characterized via enrichment analysis^23^,^24^. The principal idea behind enrichment analysis is that if a specific class of elements with an established meaning or function is much more prevalent within the group of identified results than would be expected by chance, this suggests a non-random association between the group and the class. Such enrichment can be assigned with a p-value, and can often reveal meaningful associations despite noise within the results.

In order to characterize the identified changes, we conducted enrichment analysis on the two sets of connections that were identified as differential: the set of weakened connections, and the set of strengthened connections. Each parcel was annotated according to the lobe and hemisphere in which it was located. Lobes were identified by mapping parcel spatial centers into the TD lobe map provided with the WFU Pick Atlas Tool^51^,^52^, combined with laterality information, i.e., left (x < −6), midline (−6 < x < 6) or right (x > 6). This resulted in a unique mapping of each of the 463 parcels to one of 18 possible annotations. Consequently each connection was given a pair of annotations according to the location of the two parcels comprising it.

The Hyper-geometric cumulative distribution function (HG-CDF) was used to assess the enrichment levels of the lobe representation of identified connections. HG-CDF is given in equation 1.


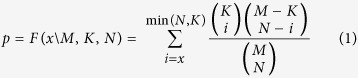


Given a population of size M, which contains K items with a desired characteristic, HG-CDF computes the probability of observing at least x items with the desired characteristic in a random sample of size N. In the current analysis, HG-CDF was calculated separately for each pair of anatomic annotations (a1 and a2), with K corresponding to the number of parcel-pairs linking a1 and a2, and x corresponding to the number of pairs that link a1 and a2 and demonstrate a significant rsFC change following stress. M and N corresponded to the number of all possible parcel-pairs, and the number of parcel-pairs demonstrating a significant rsFC change, respectively. The probabilities were corrected for multiple comparisons using Bonferroni correction.

The null hypothesis that underlies the HG-CDF is that parcel-pairs were obtained randomly and independently. As an additional filtering, to rule out dependency biases in the enrichment results, we used a random permutation test. The permutation test and associated results are described in the Supplementary section.

For each identified enrichment result, an additional measure called “enrichment factor” (EF) was calculated. For each pair of annotations (a1, a2) EF corresponds to the ratio between the relative frequency of a1– a2 links in the sample (e.g. parcel-pairs with increased rsFC) and their relative frequency in the background (i.e. all possible parcel-pairs). This descriptive measure was not used to identify the results; rather it allowed additional assessment of the extent of each of the identified enrichment results.

## Additional Information

**How to cite this article**: Maron-Katz, A. *et al.* A large-scale perspective on stress-induced alterations in resting-state networks. *Sci. Rep.* **6**, 21503; doi: 10.1038/srep21503 (2016).

## Supplementary Material

Supplementary Information

## Figures and Tables

**Table 1:** Lobe-based enrichment analysis results-.

Lobes	∆FC	^#^Connections	Corrected p-value	% of connections	enrichment factor
Temporal L; Thalamus	↑	17	1.13E-08	9%	60.1
Temporal R; Thalamus	↑	7	2.46E-05	3.7%	20.2
Parietal R; Thalamus	↑	10	4.51E-07	5.3%	47.2
Frontal R; Thalamus	↑	12	6.94E-08	6.3%	28.3
Temporal L; Parietal R	↓	30	5.76E-08	10%	8.88
Temporal R; Parietal L	↓	27	6.04E-08	9%	7.83

Results of lobe-based enrichment analysis of significantly strengthened and weakened connections (p-value <  = 0.05, Bonferroni corrected).

The enrichment factor is the ratio between the fraction of pairs with the specified lobe representation in the set of increased/decreased ∆FC pairs, and that fraction in the set of all possible connections. R = right, L = left.
