# Supplementary material for: A large-scale perspective on stress-induced alterations in resting-state networks
Source: Sci Rep. 2016 Feb 22;6:21503. doi: 10.1038/srep21503 (PMC4761902; doi:10.1038/srep21503)
Supplement: Supplementary Information [file srep21503-s1.pdf]

Supplementary information

A large-scale perspective on stress-induced alterations in resting-state networks

Adi Maron-Katz [a,b,c], Sharon Vaisvaser[a,b,d], Tamar Lin[a,e], Ron Shamir* [c,d], Talma Hendler* [a,b,d,e]

a. Functional Brain Center, Tel Aviv Sourasky Medical Center, 6 Weizmann Street, Tel Aviv 64239, Israel

b. Physiology and Pharmacology Department, Sackler Faculty of Medicine, Tel Aviv University, Tel Aviv 69978, Israel

c. Blavatnik School of Computer Science, Tel-Aviv University, Tel Aviv 69978, Israel

d. Sagol School of Neuroscience, Tel Aviv University, Tel Aviv 69978, Israel

e. School of Psychological Sciences, Tel Aviv University, Tel Aviv 69978, Israel

* These authors contributed equally to this work

Brain regions constituting enriched patterns

Table S1 contains information on parcel-pairs that were both modulated by the task (demonstrated differential rsFC following stress induction) and link enriched lobe pairs.

Validating enrichment analysis results based on a random permutation test

Since the null hypothesis that underlies the HG-CDF is that parcel pairs were obtained randomly and independently, we used a random permutation test to rule out dependency biases in the enrichment results. For this we permuted the parcel annotations while preserving the graph structure, and counted the number n of connections between pairs of annotations that passed the first (HG-CDF) test. This process was repeated 10000 times. The fraction of times in which n exceeded the observed value was used as the empirical p-value. Results of permutation test are presented in Table S2. This filter caused the exclusion of four out of 10 enrichment results identified using the HG-CDF test.

rsFC modulations sensitive to inter-individual differences in cortisol response

As previously reported by Vaisvaser et al. [1], in accordance with stress literature, two distinct cortisol groups emerged in response to stress: "*responders*", who were defined by an increase of at least 1.5 nmol/L and a 15% rise from pre-stress levels (suggested earlier in [2-4]) (n = 21); and "*non-responders*", who showed no change or diminished cortisol level (n = 33). Notably, three of the participants were not included in any of the groups due to insufficient saliva samples. For the "responders" group, an increased cortisol level was demonstrated 20 min following stress-induction (SR4) relative to all other time points (Tukey's HSD p< 0.001), as well as immediately after stress induction (SR3) relative to control (SR2, p < 0.01). The means and standard deviation (in parenthesis) of SR1, SR2, SR3, and SR4 (in nmol\ lL) for the *responders* group were 7.23 (2.1), 6.56 (1.75), 8.14 (3.23) and 10.25 (3.92), respectively.

Means and standard deviation for the *non-responders* group were 6.57(4.54), 5.91 (4.15), 5.13 (3.6) and 4.89 (3.06) (Figure S1-b).

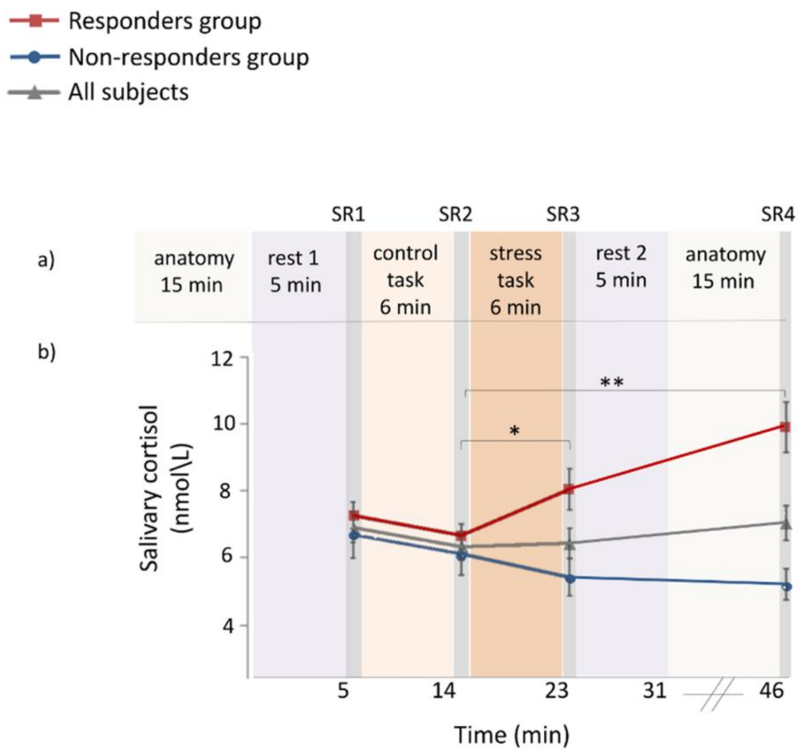

FigureS1: Cortisol response to stress on experimental timeline.
Salivary cortisol measures (b) are presented in reference to the time course of the experiment (a). Time 0 indicates the start of the first rest condition. The orange columns represent control and stress tasks (6 min each), violet columns represent 'rest' conditions (fixation, open eyes, 5 min) and light gray columns represent anatomical scans (15 min each). Between scans (dark gray columns), subjective ratings of stress and salivary cortisol samples [SR(1–4)] were collected. Bars indicate standard error.
∗ $p<0.01$, ∗∗ $p<0.001$ – Tukey's HSD post hoc.

In order to identify functional connections for which the change induced by stress was associated with cortisol increase, we used the above cortisol response-based group partition, and applied a two-sample t-test on ΔFC values of enrichment-inducing parcel-pairs. No significant inter-group difference was identified in ΔFC of any of the pairs separately (FDR q>0.4). Additionally, there was no significant inter-

group difference in the mean ΔFC magnitude of all 103 enrichment-inducing pairs (p>0.5). Following this lack of association, we conducted a similar two-sample t-test on the entire set of 106,953 parcel-pairs in the data. Once again, an FDR procedure was used to correct for multiple hypothesis testing. This analysis did not reveal any significant findings, however, a single parcel pair demonstrated a marginally significant difference in rsFC change (q=0.065, t=-5.7). This connection links two parcels centered in the posterior temporal sulcus (66,-42, 12) and in the precuneus (0,-54, 63) (Figure L2-a). Furthermore, rsFC change between these parcels correlated with the change in reported level of subjective stress following the task (SR3-SR1) across subjects (Spearman r=0.389, p=0.003). A repeated measures ANOVA applied on the rsFC values of this connection revealed a significant interaction between group and time [F(1.52)=32.47, p<0.000001]. Tuke's HSD post hoc analysis revealed an inter-group difference in rsFC of is already evident during "rest 1", where a higher positive correlation exists among the "non-responders" group (p< 0.02). Following stress, rsFC between these parcels increased among the "non-responders" group (p<0.001) and decreased among the "responders" group (p<0.005). The means and standard deviation (in parenthesis) of "rest1" and "rest2" were 0.08 (0.25) and 0.05 (0.25) respectively, for all subjects, -0.05 (0.27) and 0.14 (0.25) for the "responders" group and 0.15 (0.26) and -0.01 (0.24) for the "non-responders" group. Results are shown in Figure S2-b.

a)

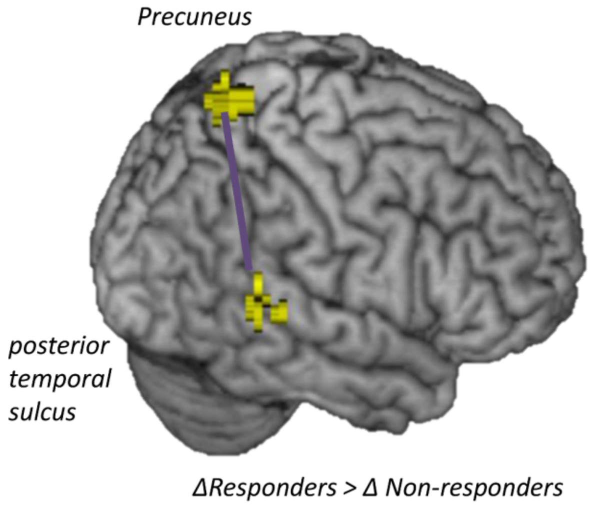

b)

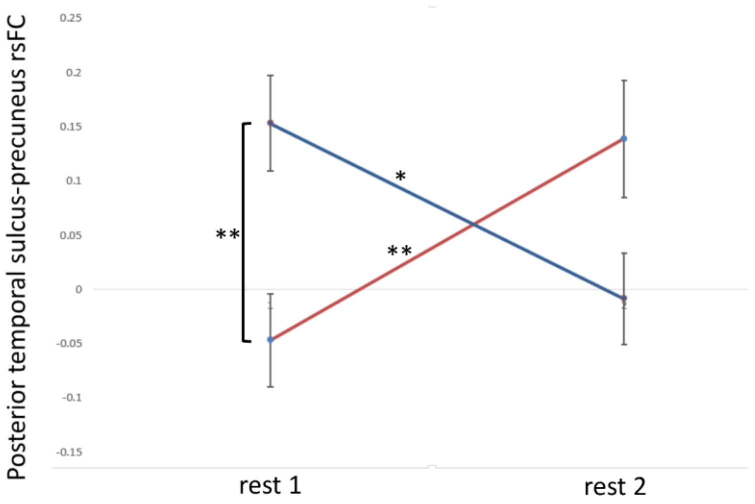

Figure S2: Result of cortisol-group based rsFC analysis

A single parcel pair (a) demonstrated a marginally significant inter-group difference in rsFC change following stress (FDR q=0.05). rsFC values are shown in (b) for the "responders" group (red) and the "non responders" group (blue). Error bars indicate standard error. *p<0.05, **p<0.005

Table S1: Enrichment-inducing parcel pairs:

A specification of all parcels-pairs that demonstrated differential rsFC following stress, and link enriched lobe pairs. Rows are sorted according to t-values.

Parcel1	X	y	z	Parcel2	x	y	z	p-value	Fdr q-value	t-value
453_	54	-9	-15	524_	-39	-51	54	2.28E-07	0.007	-5.892
93_	-45	-36	54	453_	54	-9	-15	4.86E-07	0.007	-5.688
93_	-45	-36	54	188_	63	-18	-12	7.78E-07	0.007	-5.561
196_	51	-24	54	258_	-60	-9	-24	9.79E-07	0.007	-5.499
11_	48	-27	42	102_	-54	-33	0	1.55E-06	0.01	-5.374
196_	51	-24	54	399_	-60	-12	-15	3.13E-06	0.014	-5.181
11_	48	-27	42	258_	-60	-9	-24	3.24E-06	0.014	-5.171
3_	63	-12	-21	93_	-45	-36	54	6.17E-06	0.015	-4.992
102_	-54	-33	0	508_	54	-18	45	7.02E-06	0.015	-4.955
3_	63	-12	-21	549_	-60	-15	30	7.72E-06	0.016	-4.929
158_	-54	-24	-3	233_	27	-45	66	1.08E-05	0.018	-4.835
79_	39	-36	45	102_	-54	-33	0	1.17E-05	0.018	-4.811
158_	-54	-24	-3	345_	63	-12	18	1.31E-05	0.019	-4.781
258_	-60	-9	-24	508_	54	-18	45	1.49E-05	0.019	-4.744
151_	-12	-66	57	453_	54	-9	-15	1.56E-05	0.019	-4.731
258_	-60	-9	-24	393_	39	-39	57	1.90E-05	0.02	-4.675
37_	-42	-36	42	453_	54	-9	-15	1.95E-05	0.02	-4.667
399_	-60	-12	-15	508_	54	-18	45	2.09E-05	0.02	-4.647
3_	63	-12	-21	180_	-54	-27	42	2.10E-05	0.02	-4.645
102_	-54	-33	0	345_	63	-12	18	2.36E-05	0.021	-4.612
393_	39	-39	57	399_	-60	-12	-15	2.55E-05	0.022	-4.59
102_	-54	-33	0	286_	63	-21	30	2.66E-05	0.022	-4.578
192_	-54	-3	-18	508_	54	-18	45	3.05E-05	0.024	-4.538
11_	48	-27	42	158_	-54	-24	-3	3.18E-05	0.024	-4.526
102_	-54	-33	0	393_	39	-39	57	4.12E-05	0.028	-4.451
11_	48	-27	42	341_	-54	-48	6	4.63E-05	0.028	-4.417
140_	-57	-18	-24	196_	51	-24	54	4.71E-05	0.028	-4.412
3_	63	-12	-21	151_	-12	-66	57	4.87E-05	0.028	-4.402
11_	48	-27	42	399_	-60	-12	-15	4.93E-05	0.028	-4.399
151_	-12	-66	57	496_	36	-39	-21	5.87E-05	0.031	-4.348
265_	-54	-42	45	453_	54	-9	-15	6.05E-05	0.031	-4.339
79_	39	-36	45	258_	-60	-9	-24	6.66E-05	0.033	-4.31
158_	-54	-24	-3	508_	54	-18	45	6.67E-05	0.033	-4.31
498_	63	-3	-18	549_	-60	-15	30	6.76E-05	0.033	-4.306
188_	63	-18	-12	219_	-57	-9	15	8.51E-05	0.036	-4.238
151_	-12	-66	57	326_	27	-39	-18	8.52E-05	0.036	-4.237
192_	-54	-3	-18	196_	51	-24	54	9.48E-05	0.037	-4.206

180_	-54	-27	42	316_	54	-3	-27	9.49E-05	0.037	-4.205
93_	-45	-36	54	316_	54	-3	-27	9.89E-05	0.037	-4.193
214_	-30	-42	63	264_	63	-15	3	9.97E-05	0.038	-4.191
180_	-54	-27	42	453_	54	-9	-15	0.000102	0.038	-4.183
56_	-57	-60	9	79_	39	-36	45	0.000103	0.038	-4.18
11_	48	-27	42	192_	-54	-3	-18	0.000112	0.039	-4.157
3_	63	-12	-21	37_	-42	-36	42	0.000124	0.04	-4.125
318_	-27	-57	57	453_	54	-9	-15	0.000128	0.041	-4.116
93_	-45	-36	54	498_	63	-3	-18	0.000132	0.041	-4.106
79_	39	-36	45	325_	-51	6	-27	0.000134	0.041	-4.101
11_	48	-27	42	145_	-63	-27	3	0.000137	0.041	-4.096
158_	-54	-24	-3	286_	63	-21	30	0.00015	0.043	-4.067
219_	-57	-9	15	498_	63	-3	-18	0.000156	0.043	-4.056
188_	63	-18	-12	549_	-60	-15	30	0.00017	0.045	-4.03
3_	63	-12	-21	524_	-39	-51	54	0.000173	0.045	-4.024
303_	-66	-39	-3	393_	39	-39	57	0.000178	0.045	-4.017
63_	-27	-72	36	288_	54	9	-12	0.000191	0.047	-3.995
102_	-54	-33	0	233_	27	-45	66	0.0002	0.048	-3.98
180_	-54	-27	42	498_	63	-3	-18	0.000215	0.049	-3.959
19_	-21	-78	42	288_	54	9	-12	0.000217	0.049	-3.955
72_	-60	-12	3	242_	6	-9	6	0.000228	0.05	3.941
45_	12	-63	21	420_	3	-18	9	0.000224	0.05	3.945
242_	6	-9	6	550_	63	-27	12	0.000223	0.05	3.947
248_	60	0	18	420_	3	-18	9	0.00021	0.049	3.965
24_	-6	-6	9	204_	54	0	45	0.000204	0.048	3.974
24_	-6	-6	9	173_	-66	-39	9	0.000201	0.048	3.979
35_	-3	-3	3	248_	60	0	18	0.0002	0.048	3.981
196_	51	-24	54	242_	6	-9	6	0.000183	0.046	4.008
11_	48	-27	42	242_	6	-9	6	0.000141	0.041	4.087
242_	6	-9	6	345_	63	-12	18	0.000138	0.041	4.094
159_	51	-30	21	242_	6	-9	6	0.000127	0.041	4.117
158_	-54	-24	-3	242_	6	-9	6	0.000123	0.04	4.129
24_	-6	-6	9	196_	51	-24	54	0.000119	0.04	4.139
102_	-54	-33	0	242_	6	-9	6	0.000107	0.038	4.169
35_	-3	-3	3	152_	51	-9	36	0.000106	0.038	4.173
264_	63	-15	3	420_	3	-18	9	8.79E-05	0.036	4.228
89_	54	-33	0	420_	3	-18	9	8.48E-05	0.036	4.239
229_	-48	-30	9	420_	3	-18	9	8.48E-05	0.036	4.239
28_	54	6	33	242_	6	-9	6	8.33E-05	0.035	4.244
35_	-3	-3	3	508_	54	-18	45	8.14E-05	0.035	4.251
242_	6	-9	6	264_	63	-15	3	7.93E-05	0.035	4.259
115_	54	-15	15	242_	6	-9	6	6.62E-05	0.033	4.312
24_	-6	-6	9	501_	-45	-60	21	6.59E-05	0.033	4.314
103_	54	-24	-3	242_	6	-9	6	5.72E-05	0.031	4.355

35_	-3	-3	3	68_	63	-6	30	5.50E-05	0.03	4.367
145_	-63	-27	3	242_	6	-9	6	5.35E-05	0.03	4.375
152_	51	-9	36	242_	6	-9	6	4.52E-05	0.028	4.424
24_	-6	-6	9	72_	-60	-12	3	3.07E-05	0.024	4.536
242_	6	-9	6	321_	63	-30	0	2.35E-05	0.021	4.613
102_	-54	-33	0	420_	3	-18	9	2.00E-05	0.02	4.66
24_	-6	-6	9	152_	51	-9	36	1.90E-05	0.02	4.674
24_	-6	-6	9	145_	-63	-27	3	1.69E-05	0.019	4.708
103_	54	-24	-3	420_	3	-18	9	1.48E-05	0.019	4.746
24_	-6	-6	9	102_	-54	-33	0	1.44E-05	0.019	4.753
242_	6	-9	6	413_	60	-3	6	1.40E-05	0.019	4.761
145_	-63	-27	3	420_	3	-18	9	7.28E-06	0.015	4.946
24_	-6	-6	9	229_	-48	-30	9	6.74E-06	0.015	4.967
72_	-60	-12	3	420_	3	-18	9	6.09E-06	0.015	4.995
158_	-54	-24	-3	420_	3	-18	9	5.82E-06	0.015	5.008
24_	-6	-6	9	158_	-54	-24	-3	5.51E-06	0.015	5.023
24_	-6	-6	9	68_	63	-6	30	5.17E-06	0.015	5.041
68_	63	-6	30	242_	6	-9	6	4.05E-06	0.014	5.109
242_	6	-9	6	508_	54	-18	45	3.84E-06	0.014	5.124
24_	-6	-6	9	508_	54	-18	45	3.12E-06	0.014	5.181
24_	-6	-6	9	269_	-60	-48	21	8.23E-07	0.007	5.546
242_	6	-9	6	248_	60	0	18	2.27E-07	0.007	5.893

Table S2: Permutation test results

Results of permutation test validation on lobe-enrichment patterns identified using HG-CDF test in strengthened and weakened connections. Permutation-based p-values were corrected for multiple testing using the Bonferroni correction.

Lobes	ΔFC	HG-CDF based	Permutation-	Permutation- based
**Results accepted by the permutation test**				
Temporal L; Thalamus	↑	1.13E-08	<0.0001	<0.0153
Thalamus; Parietal R	↑	2.46E-05	<0.0001	<0.0153
Temporal R; Thalamus	↑	4.51E-07	0.0001	0.0153
Frontal R; Thalamus	↑	6.94E-08	<0.0001	<0.0153
Temporal L; Parietal R	↓	5.76e-08	<0.0001	<0.0153
Temporal R; Parietal L	↓	6.04e-08	<0.0001	<0.0153
**Results rejected by the permutation test**				
Frontal L; Thalamus	↑	0.02	0.0047	0.7191
Limbic M; Parietal R	↑	0.0374	0.0027	0.4131
Frontal L; Parietal R	↓	0.012	0.0036	0.551
Frontal L; Frontal M	↓	0.0036	0.0165	1

References

1 Vaisvaser, S. *et al.* Neural traces of stress: cortisol related sustained enhancement of amygdala-hippocampal functional connectivity. *Frontiers in Human Neuroscience* **7**, doi:10.3389/fnhum.2013.00313 (2013).

2 Fehm-Wolfsdorf, G. *et al.* Auditory reflex thresholds elevated by stress-induced cortisol secretion. *Psychoneuroendocrinology* **18**, 579-589, doi:10.1016/0306-4530(93)90035-J.

3 Lupien, S. *et al.* Stress-Induced Declarative Memory Impairment in Healthy Elderly Subjects: Relationship to Cortisol Reactivity1. *The Journal of Clinical Endocrinology & Metabolism* (2013).

4 Schwabe, L., Bohringer, A., Chatterjee, M. & Schachinger, H. Effects of pre-learning stress on memory for neutral, positive and negative words: Different roles of cortisol and autonomic arousal. *Neurobiology of Learning and Memory* **90**, 44-53, (2008).
